# Novel Methotrexate-Ciprofloxacin Loaded Alginate-Clay Based Nanocomposite as Anticancer and Antibacterial Co-Drug Delivery System

**DOI:** 10.34172/apb.2021.055

**Published:** 2020-07-01

**Authors:** Mehrdad Mahkam, Fatemeh Bazmi Zeynabad, Effat Alizadeh, Mahdi Rahimi, Fariborz Rahimi, Roya Salehi

**Affiliations:** ^1^Chemistry Department, Azarbaijan Shahid Madani University, Tabriz, Iran.; ^2^Department of Medical Biotechnology, Faculty of Advanced Medical Sciences, Tabriz University of Medical Sciences, Tabriz, Iran.; ^3^Department of Organic and Biochemistry, Faculty of Chemistry, University of Tabriz, Tabriz, Iran.; ^4^Department of Electrical Engineering, University of Bonab, Bonab, Iran.; ^5^Drug Applied Research Center and Department of Medical Nanotechnology, Faculty of Advanced Medical Sciences, Tabriz University of Medical Sciences, Tabriz, Iran.

**Keywords:** Drug delivery system, Alginate, Nanoclay, PH-sensitive nanocomposite, Cancer therapy, Antibacterial activity

## Abstract

***Purpose:*** In last decades, by increasing multi-drug resistant microbial pathogens an urgent demand was felt in the development of novel antimicrobial agents.

***Methods:*** Promising nanocomposites composed of clay/alginate/imidazolium-based ionic liquid, have been developed via intercalation of calcium alginate and ionic liquid by ion exchange method. These tailored nanocomposites were used as nanocarriers to simultaneously deliver methotrexate (MTX), and ciprofloxacin (CIP), as anticancer and antibacterial agents, respectively to MCF-7 breast cancer cells. Nanocomposites were fully characterized by scanning electron microscopy studies (SEM), X-ray diffraction (XRD), Fourier transforms infrared (FTIR) spectroscopy, and thermogravimetric analysis (TGA) methods. The in vitro antimicrobial potential of the mentioned nanocomposites in free and dual-drug loaded form was investigated on Pseudomonas aeruginosa and Escherichia coli bacteria. The antitumor activity of nano-formulations was evaluated by both MTT assay and cell cycle arrest.

***Results:*** The dual drug-loaded nanocomposites with exceptionally high loading efficiency (MTX: 99 ±0.4% and CIP: 98 ±1.2%) and mean particle size of 70 nm were obtained with obvious pH-responsive MTX and CIP release (both drugs release rate was increased at pH 5.8 compared to 7.4). The antibacterial activity of CIP-loaded nanocomposites was significantly higher in comparison with free CIP (*P* <0.001). The antitumor activity results revealed that MTX cytotoxicity on MCF-7 cells was significantly higher in nano-formulations compared to free MTX (*P* <0.001). Both MTX-loaded nanocomposites caused S-phase arrest in MCF-7 cells compared to non-treated cells (*P* ˂ 0.001).

***Conclusion:*** Newly developed smart nanocomposites are potentially effective pH-sustainable delivery systems for enhanced tumor therapy.

## Introduction

Nanomedicines have been recently explored for clinical trials of cancer therapy.^1^ Nanoparticles facilitate the delivery and accumulation of drugs in tumor sites because of their capability to overcome many of the biological transport barriers^2^ and to achieve better drug targeting by enhanced permeability and retention effect.^3^ Chemical conjugation of anticancer drugs to nanoparticles which is broken by tumor-specific stimulants delivers the chemotherapeutic drugs.^4^

Chemotherapy is a common method for treatment of cancer patients but long term administration leads to drug resistance.^5,6^ To overcome drug resistance, designing new nanoparticulate drug delivery formulation based on targeted moieties has been developed recently.^7,8^ Bio-nanocomposites are biopolymer matrices (reinforced with nanoparticles such as montmorillonite) which have improved mechanical properties.^9^ In recent years, a series of biopolymers combined with clay mineral layers such as montmorillonite, hectorite, sapnotite, and laponite^10^ are progressively used in DDSs.^11,12^ Due to their unique structure and properties, laponite and montmorillonite are suitable for DDSs. However, their hydrophilic surface properties cause them not to be compatible with most polymers. Therefore, it is better to modify their surface and make the more hydrophobic.^13,14^

Alginate is a water-soluble polysaccharide, which is biocompatible, non-toxic, non-immunogenic, biodegradable and abundantly available biopolymer in nature.^15^ Alginate is used in various applications including pharmaceutical industry, food industry, drug delivery, enzyme encapsulation and contrast agent development for diagnostic imaging.^16-18^ In addition, it is an effective natural polymer which offers an attractive alternative for sustained-release systems and could change to the hydrogel form under mild pH and temperature. These advantages make alginates very useful materials for biomedical applications, especially for controlled delivery of drugs and other biologically active compounds.^18-20^ Sodium alginate undergoes ion exchange in the presence of calcium ions at room temperature. The monovalent sodium ions are replaced by divalent ions of calcium and produce a hydrogel. The alginate-based hydrogel has three-dimensional (3D) architecture in presence of divalent ions. However, ionic cross-linked alginate hydrogel has several limitations in regenerative medicine and biomedical application such as low drug loading efficiency, strength, and toughness.^21^

Methotrexate (MTX) is an anticancer drug which is generally used for treatment of many solid tumors. MTX is analogous of folic acid; MTX could act as a ligand to attach to folic acid receptor on the surface of the cancer cells such as MCF-7 cells and be uptaken in cells.^22^ It also has capability to prevent the dihydrofolate reductase enzyme activity in the cytosol of cells. MTX in lower doses is a synchronizing agent but in higher doses exhibit cytotoxic effects on cells by inhibiting DNA synthesis and finally S-phase cell cycle arrest.^23^ Cationic nanoparticles uptake, on the other hand, is higher because of the endocytosis process through interacting with the negatively charged cell membrane.^24^ Ciprofloxacin (CIP, fluoroquinolone family) is an attractive antibiotic option affecting a wide range of Gram-positive and Gram-negative organisms.^25^

During chemotherapy body’s immune system gets weak and sensitive to infectious diseases. Therefore, along with chemotherapy antibiotics are prescribed. Due to the increasing number of multi-drug resistant microbial pathogens, studies for development of novel antimicrobial agents seems to be essential. Most of antibiotics act on intracellular targets without damaging the bacterial morphology.^26,27^ There is an urgent need for the discovery of different antimicrobial agents that damage microbial membrane. Different polymers with such properties have been recently discovered.^28-32^ Samples of these antibacterial agents developed as prospective alternatives for common antibiotics are: antimicrobial peptides,^26^ synthetic cationic polymers,^33,34^ quaternized amine containing polymers,^35-38^ guanidine derivatives, polymeric guanidine salts like polyhexamethylene biguanide,^39^ antimicrobial nanoparticles and nanoclays.^40^ Mesoporous silica nanoparticles are exceptional candidates for various biomedical applications due to their straightforward synthesis, tunable pore morphologies, facile functionalization chemistries, low-toxicity degradation ways in the biological environment, and ability to carry molecular drugs and proteins in the porous core. In a study, therapeutic efficacy of silica-loaded antibiotics, in comparison with their officinal forms, on Staphylococcus aureus, Pseudomonas aeruginosa, and Escherichia coli was assessed in vitro and in experimental models of sepsis induced by various strains of microorganisms in mice (CBAxC57BL/6) F1. Their results revealed expediency of using this modification of antibiotics to boost their therapeutic efficacy in experimental sepsis.^41,42^ The advantage of the innovative antimicrobial agents is their diverse ways of action, which is different from common antibiotics that main clinical pathogens are now resistant to. Notable endeavors to produce antibacterial nanocarriers as delivery system for anticancer drugs are underway.^43,44^

In this study, a novel cationic biodegradable nanocomposites composed of clay/alginate/imidazolium-based ionic liquid was engineered for targeted MCF-7 breast cancer cells elimination. The conjugation of MTX to these biodegradable antibacterial nanoparticles with cationic charge can aid in improving the MTX cell uptake and inhibit the proliferation of cancer cells.

## Materials and Methods

### Materials

Chemical reagents including tetraethylorthosilicate (TEOS), 1-Methylimidazole (m-Im), 3-chloropropyl trimethoxysilane, ammonia solution (37%), and sodium alginate were purchased from Sigma–Aldrich Co. The solvents such as absolute methanol, ethanol, n-hexane, dimethyl sulfoxide were purchased from Merck Co. (All solvents were purified according to the standard methods). MTX and CIP were obtained from Zahravi Pharmaceuticals (Iran). Penicillin– streptomycin, amphotericin B, trypsin-EDTA, fetal bovine serum (FBS) and Roswell Park Memorial Institute (RPMI) 1640 medium were purchased from Gibco-Thermo Fisher Scientific Corporation (Waltham, Massachusetts, USA). Propidium iodide (PI), 4′,6-Diamidine-2′-phenylindole dihydrochloride (DAPI), and 3-(4, 5-dimethylthiazol-2-yl)-2, 5-diphenyltetrazolium bromide (MTT) were obtained from Sigma-Aldrich Co.

### Synthesis of silica nanoparticles (SiO_2_ NPs)

SiO_2_ NPs were prepared by sol-gel method according to the protocol reported previously by Rasouli et al.^45^ In brief, 3.96 mL (220 mmol) deionized water and 1.50 mL (20 mmol) ammonia solution were mixed. Subsequently, 200 mL absolute methanol was added to the above solution under vigorous stirring. To obtain SiO_2_ NPs, 20.82 g TEOS (1000 mmol) was added dropwise at ambient temperature and reaction was continued for three days. The resulting suspension was precipitated with n-hexane and centrifuged to obtain white SiO_2_ NPs. The powder was freeze-dried and used for the next step.

### Synthesis of cationic ionic liquid-modified SiO_2_ NPs (ImIL-MSNs)

Imidazolium-based ionic liquid (ImIL) was synthesized according to the protocol explained in our previous work.^46^ At the beginning 1-methylimidazole (1.03 g; 12.54 mmol) was poured in a three necked round bottom flask equipped with dropping funnel and refluxing system. Subsequently, CPMTS (3.02 g; 12.54 mmol) was added dropwise via dropping funnel. The resulting solution was refluxed for three days at 80°C under the nitrogen flow. After completion of the reaction, the obtained mixture was purified through multiple washing with THF. The residual THF was evaporated at vacuum oven at 50°C for 48 hours. 1-methyl-3-(3-trimethoxysilylpropyl) imidazolium chloride (Im-IL) with a honey-like appearance was obtained with high yield. In the second step, SiO_2_ NPs (1.016 g) and Im-IL (0.30 g, 0.929 mmol) were added in 5 mL CH_2_Cl_2_ and stirred at ambient temperature for three days. The obtained NPs were allowed to settle down and decanted. The modified SiO_2_ NPs were extracted with CH_2_Cl_2_ prior to being dried for several hours in the vacuum oven.

### Synthesis of organo-modified nanoclays Laponite-RD (OLP) and montmorillonite (OMT)

The organo-modified Laponite-RD (OLP) and organo-modified montmorillonite (OMT) nanoclays were prepared separately according to the following procedure. One gram of nanoclay (Laponite-RD or sodium montmorillonite) and 1.1 g of ImIL-MSNs was added in 1 liter of deionized water and heated to 50°C. To obtain homogenous suspension, the mixture was sonicated for 5 minutes by a probe-type ultrasonic generator (400 W). The resulting suspension was mixed for three days. Finally, the suspension was washed with hot deionized water to remove all chloride ions which then can be detected with silver nitrate solution (0.1 N). The resulted OLP or OMT was dried under vacuum condition at 50°C.

### Synthesis of OLP–alginate (OLPALg) and OMT-alginate (OMTALg) nanocomposites

Half a gram of OLP or OMT was added into 15 ml deionized water and dispersed for 1 hour. Then, 0.5 g of sodium alginate was added and dispersed for five minutes with the aid of a probe-type ultrasonic generator (400 W) and stirred for 48 hours. After that, CaCl_2_ solution (10 wt%) was added. Finally, the solution was freeze-dried and OLP–Alginate (OLPALg) and OMT-Alginate (OMTALg) nanocomposites were obtained.

### Loading and release studies of MTX and CIP

*Loading study of MTX and CIP:*300 mg of nanocomposite was ultrasonically dispersed in 10 mL MTX solution (2 mg mL-^1^) for 3 min. The suspension was shaken by magnetic stirrer at ambient temperature overnight. Then, 10 mg CIP was added to the suspension and stirred at room temperature for 24 h. MTX-CIP loaded nanocomposite was separated from free MTX-CIP solution through centrifuge with Amicon filter of nominal molecular weight limit of 30 kDa at 5000 rpm for 30 min and finally freeze dried to get dry powder.

*Release study of MTX and CIP*: appropriate amount of MTX-CIP loaded nanocomposite was dispersed in 2 mL phosphate buffered saline (PBS) solution. Subsequently, the suspension was transferred into a dialysis bag (MWCO 12000 Da, Sigma-Aldrich) and immersed in 8 mL PBS solution with different pH values of 4.0, 5.8 and 7.4. This system was placed in a Heidolph shaking incubator which is set at 100 rpm and 37°C during the release study. At the appropriate time intervals, the whole solution was taken out and replaced with equal amount of fresh PBS solution. The amount of MTX and CIP in the release medium was measured by HPLC-UV method at the wavelength of 303 and 267 nm, respectively.^46,47^ All measurements were done in triplicate. MTX and CIP loading and encapsulation efficiencies, and released amount were calculated by the following formulas:

(1) $$\text{Drug Loading capacity }\left(\text{\%},\frac{\text{w}}{\text{w}}\right)=\frac{\text{Mass of drug in nanocomposite}}{\text{Mass of nanocomposite}}\times 100\;$$

(2) $$\text{Drug encapsulation efficiency}\left(\text{\%},\frac{\text{w}}{\text{w}}\right)=\frac{\text{Mass of drug in nanocomposite}}{\text{Mass of feed drug}}\times 100\;$$

(3) $$\text{Drug release }\left(\text{\%}\right)=\;\frac{\text{ammount of drug release in medium}}{\text{amount of drug loaded in nanocomposite}}\;\times 100\;$$

### Instrumentation

#### Fourier transforms infrared (FTIR) spectroscopy

To determine the chemical structures of the Laponite-RD, montmorillonite, ImIL-MSNs, OLP, OLPALg nanocomposite, OMT, and OMTALg nanocomposites, FTIR spectroscopy (Equinox 55 LS 101, Bruker, Germany) was used. Firstly, sample’s disks were made with KBr at the ratio of 1 to 50 (sample to KBr). The prepared disks were scanned against a blank KBr disk at wavenumber ranging from 500 to 4000 cm-^1^.

#### X-ray diffraction (XRD)

Powder XRD graphs of the Laponite-RD, montmorillonite, OLP, OLPALg nanocomposite, OMT, and OMTALg nanocomposite were recorded on a Bruker AXS model D8 Advance Diffractometer using CuKα radiation (λ=1.542A°), with the Bragg angle ranging from 2θ= 3–70°.

#### Scanning electron microscopy studies (SEM)

Morphology and average particle size of OLP, OLPALg nanocomposite, OMT and OMTALg nanocomposite were measured by a field emission scanning electron microscope-energy dispersive using X-ray (FESEM-EDX), TESCAN MIRA3, Czech Republic. Particle size was obtained by measuring the diameters of at least 300 particles shown in SEM using image analysis software (Image-Pro plus 4.5; Media Cybernetics, Silver Spring, USA).

#### Thermogravimetric analysis (TGA)

TGA of Laponite-RD, montmorillonite, OLP, OMT, OLPALg and OMTALg nanocomposites were performed with a Mettler-Toledo model 822 instrument. TGA disintegration patterns were obtained under an inert atmosphere (N_2_) at a heating rate of 10°C per minute from 50 to 850°C.

### Preparation of inoculum

The standard strain of *Pseudomonas aeruginosa*(ATCC: 25922*)*and *Escherichia coli*(ATCC: 27853) were received in lyophilized form from Pasture Institute of Iran, Tehran, Iran. These strains were activated by culturing in sterile nutrient agar (Liofilchem, Italy) for 48 hours at 37°C. A single colony from grown plate was transferred into nutrient broth and incubated over night at 37°C. After incubation time, they were harvested by centrifugation at 1100 g for 10 minutes and rinsed two times and re-suspended in Ringer solution to provide an optical density of around 0.1 at 540 nm (bacterial concentration around 10^8^ CFUmL-^1^) with a spectrophotometer (Coleman, USA).^29^

### Antimicrobial activity

The antimicrobial activities of prepared OLPALg, OMTALg, free CIP, CIP-loaded OLPALg and CIP-loaded OMTALg nano-formulations were evaluated by minimum inhibitory concentration (MIC) method by serial dilution against microbial strains, *P. aeruginosa* and *E. coli* as standard strains. Briefly *E. coli*and *P. aeruginosa* bacterial inoculum (concentrations equal to 0.5 McFarland standards) in Muller-Hinton Broth medium were mixed with serially diluted free CIP, CIP-loaded OLPALg and CIP-loaded OMTALg nano-suspension. The mentioned samples were treated with various concentrations in the range of 0.006-3.125 μg.mL-^1^ for* E. coli*and 0.195-12.5 μg.mL-^1^ for *P. aeruginosa* bacterial inoculum. CIP-free OLPALg and OMTALg nanocomposites were both treated with various concentrations in the range of 195 to 1×10^5^ μg.mL-^1^ with both* E. coli*and *P. aeruginosa* bacterial inoculum. After 24 hours incubation at 37°C, the first plate with no growth of bacteria was chosen as MIC. MIC evaluation was done according to the Clinical and Laboratory Standards Institute (CLSI) M27-A3 and CLSI M100-S22 (12-14). Control tubes with the Muller Hilton agar (without ginger extract) were used as a control. All examinations were performed in triplicate.

### Cell culture and in vitro cytotoxicity assay

MCF-7 cells (human breast adenocarcinoma cell line) were obtained from National Cell Bank of Iran (NCBI) affiliated to Pasteur Institute of Iran (Tehran, Iran) and cultured with the method described previously.^29^ MCF-7 cells were seeded in 96-well microplates (7 × 10^3^ cells per well) and after incubation for 24 hours, were treated with free MTX, MTX-loaded OLPALg and MTX-loaded OMTALg nanocomposites with various concentrations (5, 10, 25, 50 and 100 μg mL-^1^) as well as free nanocomposites of OMTALg and OLPALg (250, 500 and 1000 μg mL-^1^). The cytotoxicity of nanocomposites and the antitumor activity of drug loaded nanocomposites were evaluated by MTT method as previously described.^46^ The absorbance of wells was read at 570 nm with a reference wavelength of 630 nm by the ELISA plate reader (Bio-Tek Instruments, USA).

### Cell cycle analysis

For cell cycle analysis, MCF-7 cells were seeded into each well of 6 well plates (1×10^5^ cells per well) and incubated for 24 hours. Then cells were treated with free MTX or encapsulated OLPALg and OMTALg (10, 50, 100 µg/mL) as well as drug free OLPALg and OMTALg nanocomposites (100 µg/mL). Non-treated cells were considered as control group. After 72 hours, cell cycle analysis was performed as described previously.^48^ The DNA content of the cells were analyzed using Flow cytometry (Becton Dickinson Immunocytometry Systems, San Jose, CA, USA) in order to reveal population frequencies in different cell cycle phases.

### Statistical analysis

Analysis of variance (ANOVA) and Student’s *t* test was used to determine the significant differences among groups. The difference was considered statistically significant at *P* values less than 0.05. Data were shown as a mean ± standard deviation (SD).

## Results and Discussion

### Preparation of multifunctional nanocomposites

**Figure 1:**
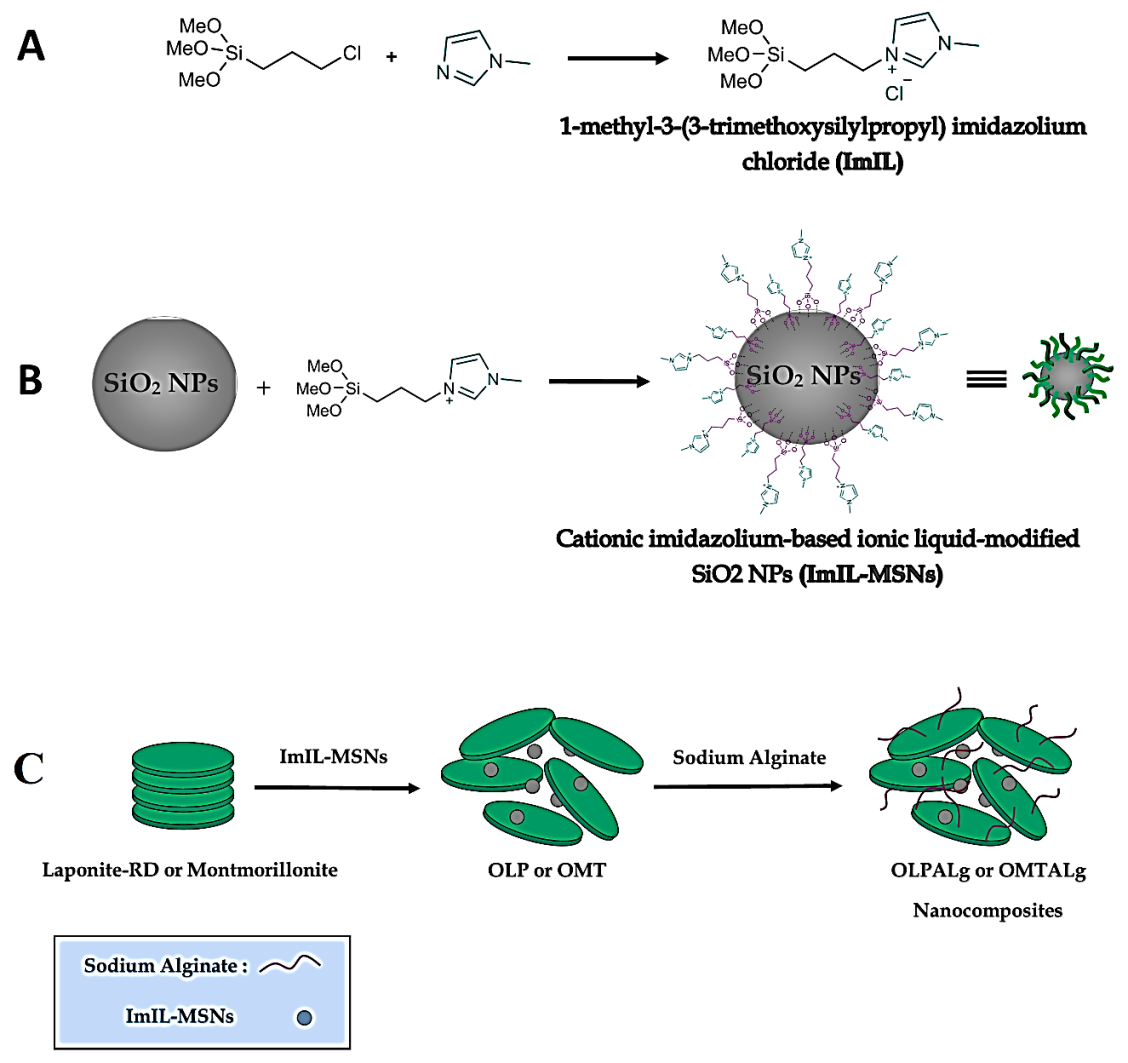
Detailed steps for the synthesis of A) ImIL, B)ImIL-MSNs and C) OLPALg and OMTALg nanocomposites.

The step by step nanocomposites’ synthesis route is shown schematically in Figure 1.

### Characterization of nanocomposites

#### FTIR spectra

**Figure 2:**
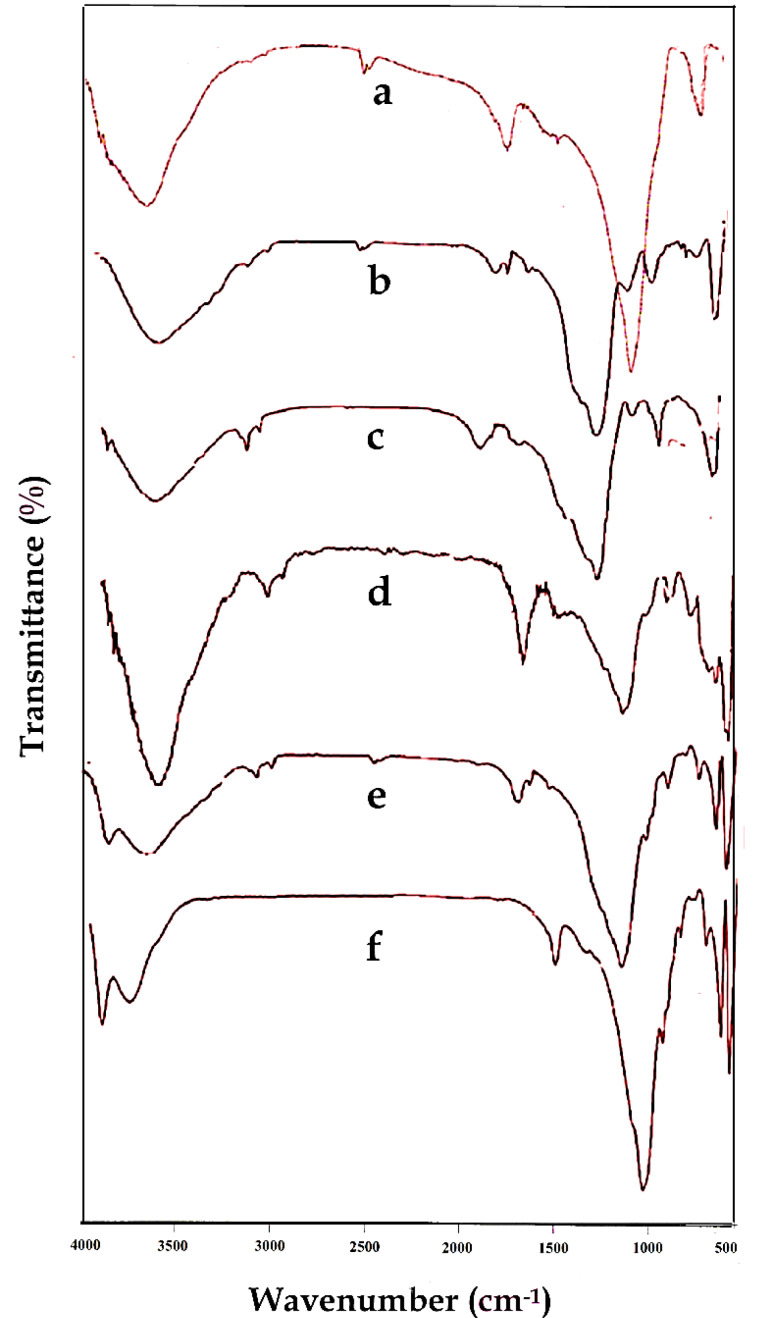
FTIR spectra of Laponite-RD (a), OLP (b), OLPALg (c), montmorillonite (d), OMT (e), and OMTALg (f).

The chemical structures of the Laponite-RD, OLP, OLPALg, montmorillonite, OMT, and OMTALg were studied by FTIR spectroscopy which is shown in Figure 2. Laponite-RD characteristic peaks appeared at 1008 and 3451 cm-^1^ (Figure 2a) which corresponds to the stretching vibration bonds of Si-O and O-H, respectively. The peak at 1634 cm-^1^ was attributed to the O-H deformation band of physisorbed water of Laponite-RD. In FTIR spectrum of OLP (Figure 2b), the broad peak at 3100-3500 cm-^1^ was attributed to stretching vibration of O-H group of silanol. The relatively weak peaks at 1638 and 1576 cm-^1^ were associated with stretching vibration of C=C aliphatic and C=C aromatic ring bonds. The strong peaks at 1088 cm-^1^ and 802 cm-^1^ were characteristic of Si-O-Si and O-H of silanols bonds. Figure 2c shows the spectrum of prepared OLPALg. The peaks at 3422 and 2924 cm-^1^ related to stretching vibration of O-H and aromatic hydrogen bonds, respectively. Strong peaks at 1637 cm-^1^ and 1524 cm-^1^ represent C=C bonds of imidazolium ring. Besides, sodium alginate showed asymmetric and symmetric stretching vibrations at 1637 cm-^1^ and 1415 cm-^1^ respectively due to carboxyl anions. Therefore, qualitative evidence of the presence of either ImIL or alginate in the interlayer space of Laponite-RD was provided by FTIR spectroscopy.

The FTIR spectrum in Figure 2d illustrates the characteristic signals of the montmorillonite. Stretching vibration bands of Si-O-Si and Si-O-Al were appeared around 1043 cm-^1^ and 531 cm-^1^, respectively. Besides, the characteristic peak at 816 cm-^1^ is attributed to the stretching vibration of Al–Al–OH moieties in the octahedral layer. The interlayer water was apparent at 3452 cm-^1^ with broad and strong intensity. The hydroxyl functional groups (-OH) of Al–OH and Si–OH were observed at 3698 cm-^1^ with broad peaks similar to water. The O-H bending mode of absorbed water was indicated as a series of overlaid bands at 1638 cm-^1^. Figure 2e shows FTIR spectrum of OMT. As seen in this spectrum, the new signals appeared which confirmed that MMT was successfully modified. The FTIR spectrum of OMTALg (Figure 2f) revealed a new peak at 1420 cm-^1^ owing to carboxyl anions and at 1049 cm-^1^ owing to oxygen stretching in cyclic ether bridge. The peak at 3449 cm-^1^ corresponds to O-H stretching vibration.

#### XRD analysis

**Figure 3:**
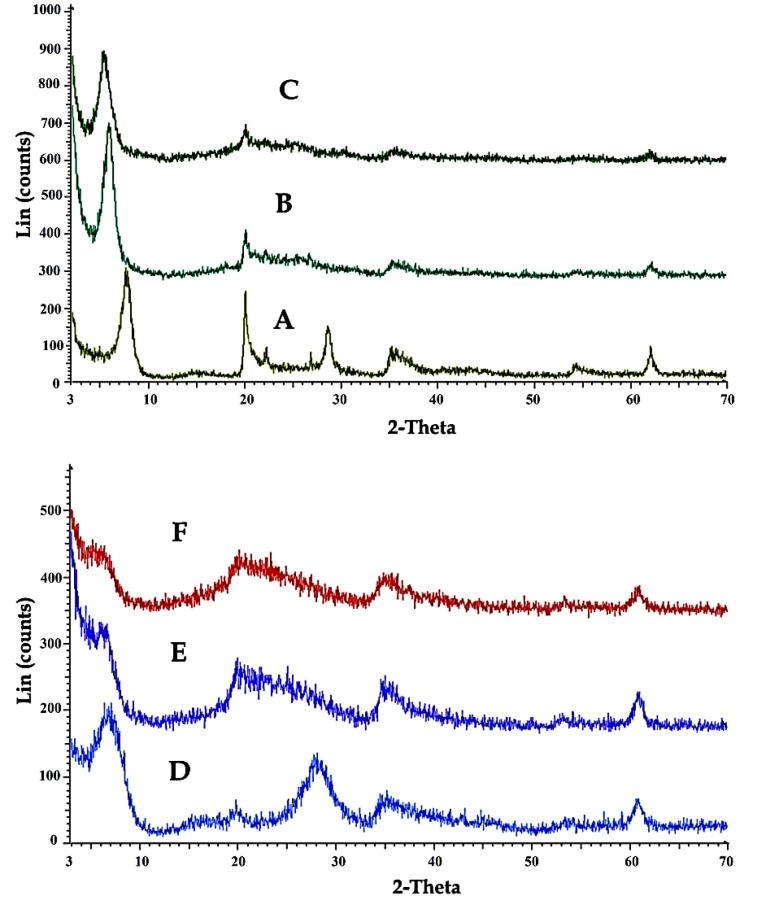
XRD patterns of Laponite-RD (A), OLP (B), OLPALg (C), montmorillonite (D), OMT (E), and OMTALg (F).

Figure 3 shows XRD patterns of Laponite-RD (A), OLP (B), OLPALg (C), montmorillonite (D), OMT (E), and OMTALg (F) nanocomposite. The changes of the basal spacing of the resulted materials reflect the intercalation of imidazolium-based ionic liquid modified silica nanoparticles and alginate into Laponite-RD interlayer spaces. The basal spacing of Laponite-RD was 12.54 nm at 2θ = 7.04°. After modification of Laponite-RD with ImIL-MSNs to yield OLP, the basal spacing of the clay increased from 12.54 to 15.32 nm at 2θ = 5.76° (Figure 3B). In XRD pattern of OLPALg as the final nanocomposite, intercalation of sodium alginate in OLP nanoclays interface was confirmed by increasing the basal spacing to 17.24 nm at 2θ = 5.11° (Figure 3C).

Our XRD analysis confirmed the intercalation of ionic liquid in montmorillonite. XRD pattern of pure montmorillonite (Figure 3D) shows a shift in the lower angle region of the peak in the 001 plane, from 2θ= 7.69° to 5.86° in modified clay (Figure 3E), resulting in an increase in the corresponding *d*-spacing from 11.48 nm to 15.05 nm. This relative increase in the *d*-spacing confirms the intercalation of ImIL-MSNs in montmorillonite (OMT). Intercalation of sodium alginate in OMT nanoclays interface (OMTALg) was confirmed by a shift in the lower angle region in the 001 plane, from 2θ= 5.86° to 5.38° resulting in an increase in *d*-spacing from 15.05 nm to 16.41 nm (Figure 3F).

According to the Bragg’s law, the peak altering from upper to lower refraction angle is related to an increase in *d*-spacing.^49^ Thus, our results confirm that ImIL-MSNs ionic liquid and alginate has been effectively intercalated into silicate layers of Laponite-RD and montmorillonite after sodium ion exchanged.

#### Surface morphology

**Figure 4:**
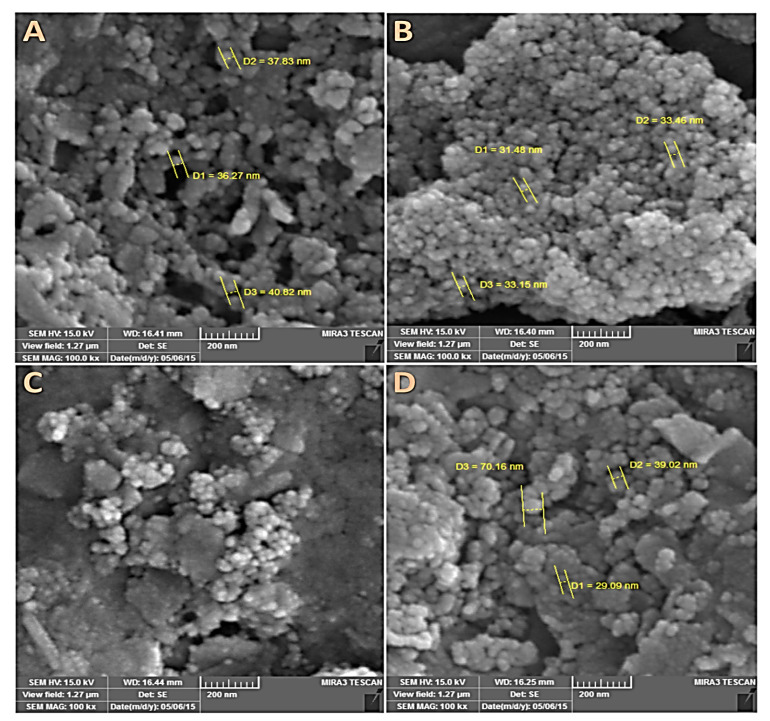
SEM images of OLP (A), OLPALg (B), OMMT (C), and OMTALg (D) nanocomposites.

The SEM technique was used to investigate the surface morphology of OLP, OLPALg, OMT, and OMTALg nanocomposites (Figure 4A-D). According to SEM images, the particles had uniform spherical morphology with size in the range of 30-70 nm. The presence of Al, Si, Mg, C, O, and N elements predicted in the structure of samples were confirmed by EDX studies. All EDX data were summarized in Table 1. The elemental analysis results confirmed successful intercalation at both nanoclays interlayers. In OLPALg and OMTALg nanocomposites the atomic ratio of Si and O atoms were increased and the atomic ratio of Al atom was decreased compared to OLP and OMT, which validated the successful intercalation of modified silica and alginate in nanoclays.

**Table 1:** Results ofEDX elemental analysis of the OLP, OLPALg, OMT and OMTALg nanocomposites

**Element**	**OLP**		**OLPALg**		**OMT**	**OMTALg**	
	**A%**	**W%**	**A%**	**W%**	**A%**	**A%**	**W%**
C	36.72	22.11	31.82	19.84	38.77	27.93	19.60
N	3.73	2.62	6.05	4.40	4.44	6.18	5.06
O	9.80	7.86	19.75	16.40	26.16	45.76	42.78
Mg	0.55	0.67	2.79	3.52	0.43	0.98	1.39
Al	45.71	61.83	33.01	46.24	22.51	4.23	6.67
Si	3.49	4.91	6.58	9.60	7.69	14.93	24.50
Total	100.00	100.00	100.00	100.00	100.00	100.00	100.00

#### Thermal analysis

**Figure 5:**
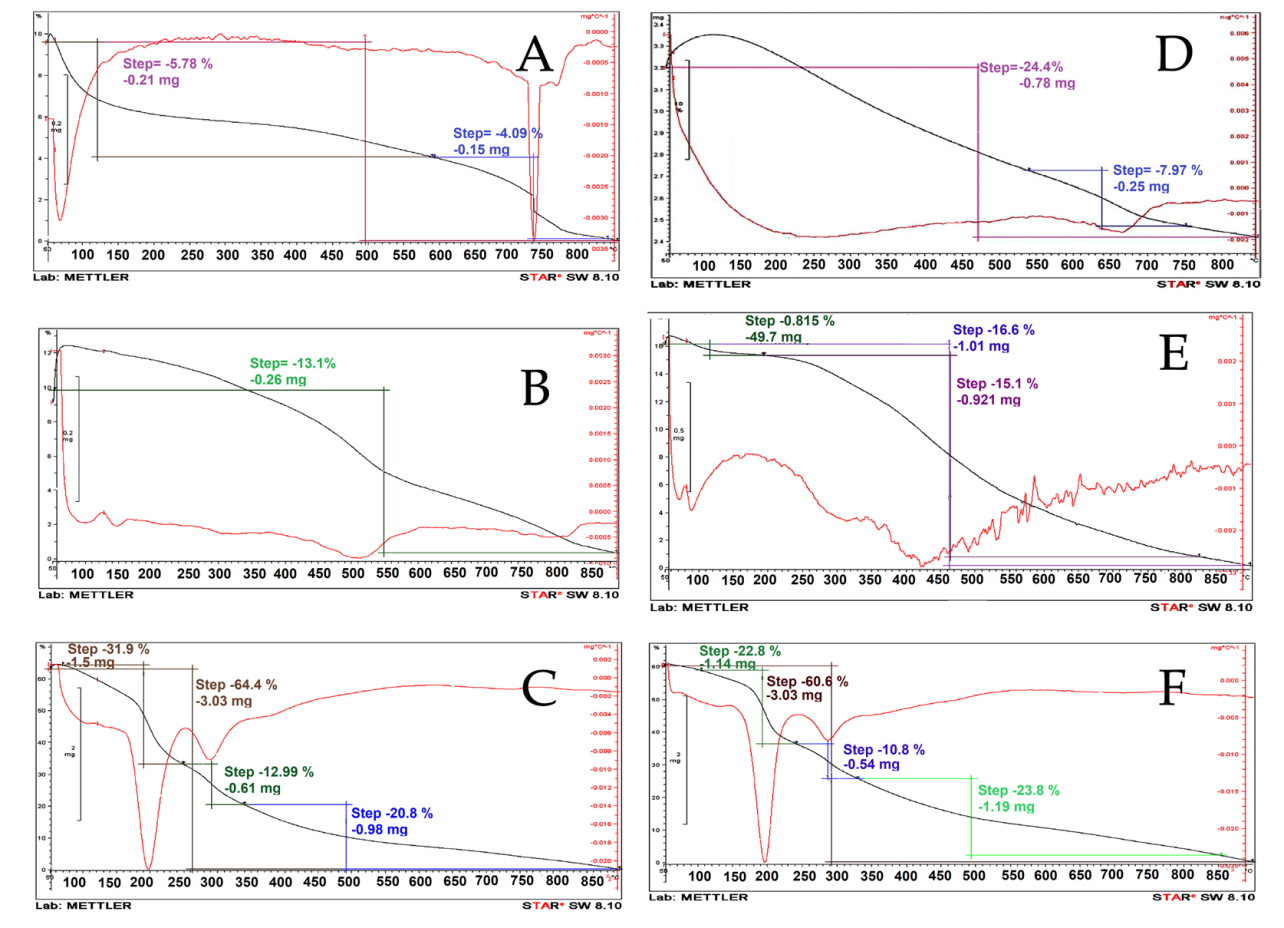
TGA analysis of Laponite-RD (A), OLP (B), and OLPALg (C), montmorillonite (D) OMT (E), and OMTALg nanocomposites (F).

Results of thermogravimetric analysis (TGA/DTG) profile for Laponite-RD, OLP, and OLPALg nanocomposite are presented in Figure 5A-C. The onset of degradation temperatures of OLP and OLPALg nanocomposites were about 550 and 200°C, respectively. The weight loss of pure clay at the temperature around 100°C corresponds to the elimination of water coordinated with Na^+^ in the interlayer. TGA thermograms of Laponite-RD exhibited the one-stage degradation behavior which is in-line with previous studies.^50,51^ TGA thermograms of OLPALg nanocomposite indicated a four-staged weight loss (below 200, 200–300, 300-500 and above 500°C). The little weight loss below 100°C is due to physically combined water. The decomposition of intercalated organic parts happened in the temperature range of 200–300°C. A weight loss between 300-500°C was related to elimination of structural hydroxyl group.^52^ The difference in TGA pattern between unmodified and modified clay proved the intercalation of clay.

TGA profile of montmorillonite, OMT, and OMTALg nanocomposite is presented in Figure 5D-F. Weight losses were observed for OMTALg nanocomposite at four different temperature regions (100–200, 200–350, 350–500 and 500–850°C). The peak at 100 to 200°C is attributed to the evaporation of adsorbed water and organic solvents. The imidazolium and alginate parts of nanocomposite were decomposed at the temperature range of 200 to 500°C with 82% weight loss that is related to the combustion of organic moiety of nanocomposite. The maximum rate degradation temperature (Tmax, first derivative peak temperature) was 250°C.

### Drug loading

OLPALg and OMTALg developed here are multifunctional nanocomposites that contain abundant functional groups with a potent ability to simultaneously interact with MTX and CIP. In aqueous dispersion, Laponite-RD and montmorillonite have multiple sites to interact with different kinds of drugs by ionic interaction and hydrogen bonding. These sites, that offer easy routes to interact with variety of drugs, include presence of exchangeable cations, O-H groups at the broken edges of the clay platelets, presence of considerable negative charge on the surfaces, and infirm positive charge on the boundaries. Drug loading experiment was performed at pH 7.4, in which the net charge of MSNs was negative (due to the de-protonation of free surface silanol groups with pka=6.8). At this pH, MTX was negative (due to the de-protonation of two carboxylic acid groups, pKa = 3.8 and 4.8)^45,46^ and CIP had both negative and positive charges (due to de-protonation of the carboxylic acid and protonation of the nitrogen on the piperazinyl ring, pKa = 6.09 and 8.74).^47^ Multitude of possibilities including electrostatic interaction, intercalation, hydrogen bonding, and physical adsorption might be involved in loading MTX and CIP to the OLPALg and OMTALg nanocomposites. The drug loading results revealed that conjugation of the first drug (MTX) in modified nanocomposites did not affect the loading capacity for encapsulation of the second drug (CIP). Excellent encapsulation efficiency was observed in simultaneous loading of MTX (99 ±0.4%) and CIP (98 ±1.2%) and loading capacity of 9.9±0.1% and 9.8±0.3%. Such high loading efficiency values of the developed nano-clay makes these nanocomposites as exceptional drug delivery candidates.

### In-vitro drug release study

**Figure 6:**
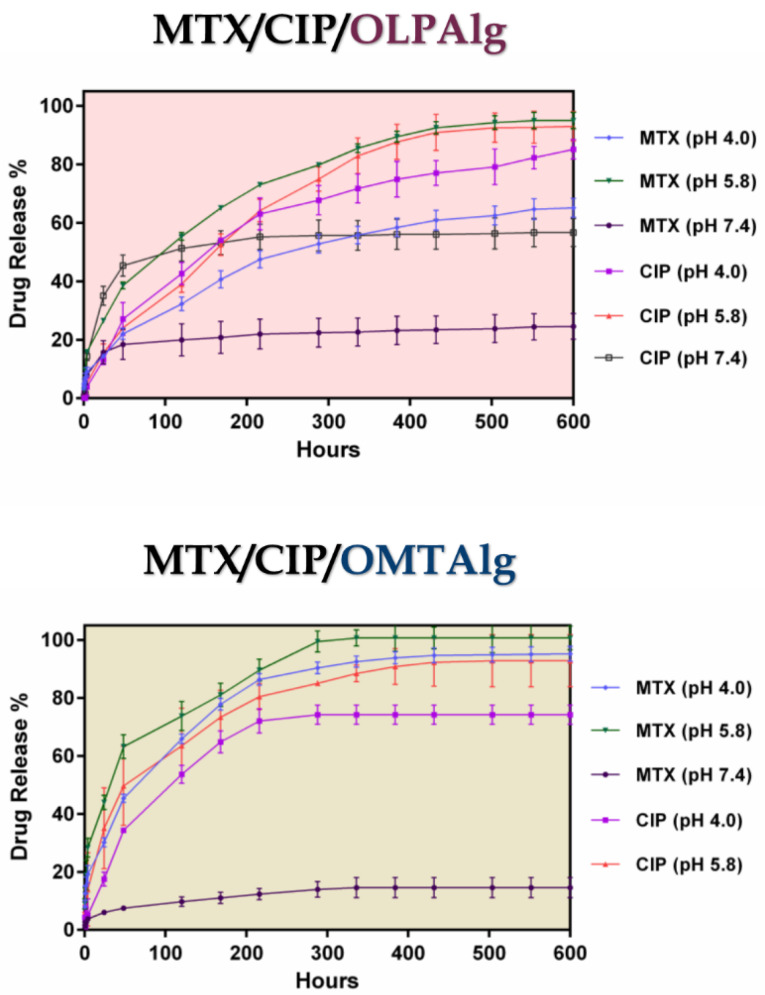
Cumulative release of MTX, and CIP at various pH values (7.4, 5.8, graph and 4.0) at 37°C for MTX-CIP loaded OLPALg (MTX/CIP/OLPALg) (upper graph) and MTX-CIP loaded OMTALg (MTX/CIP/OMTALg) (lower graph).

Figure 6 shows the pH-responsive drug release profile of MTX-CIP loaded OLPALg and OMTALg nanocomposites at three different pH values (pH 7.4, 5.8, and 4) for 25 days at 37°C. Three stages (I, II, and III) were observed for MTX and CIP release from nanocomposites. Initial burst release at first 24 hours (stage I), followed by fast release from 24-300 hours for OMTALg and 24-400 for OLPALg (stage II), and finally constant release from 300-600 h for OMTALg and 400-600 for OLPALg (stage III).

At the initial stage (stage I), the release was found to follow a diffusing manner (first 24 hours) without the significant pH-dependent release profile. The physically adsorbed drugs were also released at this stage. At stages II and III, the pH-dependent release from modified nano-clays became dominant. It was assumed that the part of MTX and CIP that loaded by pH-dependent ionic interaction and hydrogen bonding in NPs started to release in these stages. It is possible that the intercalated CIP and MTX were released and substituted with water at these stages. At both MTX-CIP loaded OMTALg and OLPALg nano-formulations, MTX and CIP release at pH 5.8 was faster than at pH 4.0. The slowest release was observed at pH 7.4 in which only 14 and 24% of MTX was released from OLPALg and OMTALg nanocomposites after 25 days, respectively. Due to the negligible CIP release at pH 7.4 from OMTALg (almost zero), this part was omitted from the Figure 6 (lower graph). Slow release of CIP and MTX at pH 7.4 was due to the strong ionic interaction between both drugs and nanocomposites as mentioned above.

The mutual interactions between the MTX/CIP and the smart OLPALg and OMTALg nanocomposites influence the differences in the release profiles. Entrapments of drugs in nanocomposites at pH 7.4 have proved due the interaction between various functional groups such as amine, hydroxyl and carboxylic acid which greatly restrict the remaining drug from releasing. By enhancing the drug/nanocomposite interaction and entrapment, the drug release rates were decreased.^53^ MTX and CIP show the degree of ionization depends on pH conditions and presence of repulsive force among CIP, MTX, and NPs at pH 5.8, cause the multilayer covering loose that resulted to faster drugs releases from the NPs. However, electrostatic interaction between MTX, CIP and the multifunctional NPs at pH 7.4 let to incomplete drug release from MTX-CIP loaded smart OLPALg and OMTALg nanocomposites at the end of the studied release profile. This could be a possible reason for depot or reservoir effect of our newly prepared nano-formulation to simultaneous release the multiple drugs in longer time intervals. This property of MTX-CIP loaded smart OLPALg and OMTALg nanocomposites reduce the side effects of drugs on normal tissues with effectively treat tumors under acidic conditions.

### In-vitro antibacterial activity of nanocomposites

The newly developed multifunctional OLPALg and OMTALg nanocomposites have a cationic segments containing imidazolium ionic liquid segments intercalated in Laponit-RD and montmorillonite. Previous studies proved antibacterial properties of different polymeric nanocomposites with cationic blocks.^28-30,32,54^ In order to investigate the probable antibacterial ability of prepared nanocomposites, their antibacterial tests were performed against the standard microbial strains of *P. aeruginosa*and *E. coli* (Table 2). The mentioned bacteria were treated with OLPALg, OMTALg, CIP/OLPALg and CIP/OMTALg nanocomposites with various concentrations. MIC values for OLPALg and OMTALg on *E. coli* strain were 781.25 and 390.62 μg mL^−1^, respectively, confirming that the developed nanoparticle had antibacterial activities. Moreover, no antimicrobial activities were observed for OLPALg and OMTALg on *P. aeruginosa* at the concentration range of 195 to 1×10^5^ μg mL^−1^. MIC values for free CIP, CIP-loaded OLPALg and CIP-loaded OMTALg nanocomposites on *P. aeruginosa* were 6.25, 1.56 and 0.78 μg·mL-^1^, respectively. MIC values for free CIP, CIP-loaded OLPALg and CIP-loaded OMTALg nanocomposites on *E. coli* were 1.56, 0.097 and 0.048 μg mL-^1^, respectively. In the control sample with no treatment, dense bacterial colonies were monitored. As a result, antibacterial activity of CIP-loaded OLPALg and OMTALg nanocomposites were increased significantly in comparison with free CIP (*P*_ value_<0.001) for both bacteria strains. The MIC value of developed novel nanocomposites as antimicrobial agents varied based on the microorganism species.

**Table 2:** Minimum Inhibitory Concentration (MIC) results of OLPALg, OMTALg, CIP-loaded OLPALg, and CIP-loaded OMTALg nanocomposites against *E.coli* and *P. aeruginosa*

	***E. coli***		***P. aeruginosa***	
	**Treated Concentrations** **(µg.mL**-^1^**)**	MIC^*^ (µg.mL-^1^**)**	**Treated Concentrations** **(µg.mL**-^1^**)**	MIC^*^ (µg.mL-^1^**)**
OLPALg	195-1×10^5^	781.25	195-1×10^5^	**-**
OMTALg	195-1×10^5^	390.62	195-1×10^5^	**-**
CIP-loaded OLPALg	0.006-3.125	0.097	0.195-12.5	1.56
CIP-loaded OMTALg	0.006-3.125	0.048	0.195-12.5	0.78
CIP	0.006-3.125	1.56	0.195-12.5	6.25

* All MIC evaluations were performed in triplicate and average results were reported as MIC.

### In-vitrocell assay

**Figure 7:**
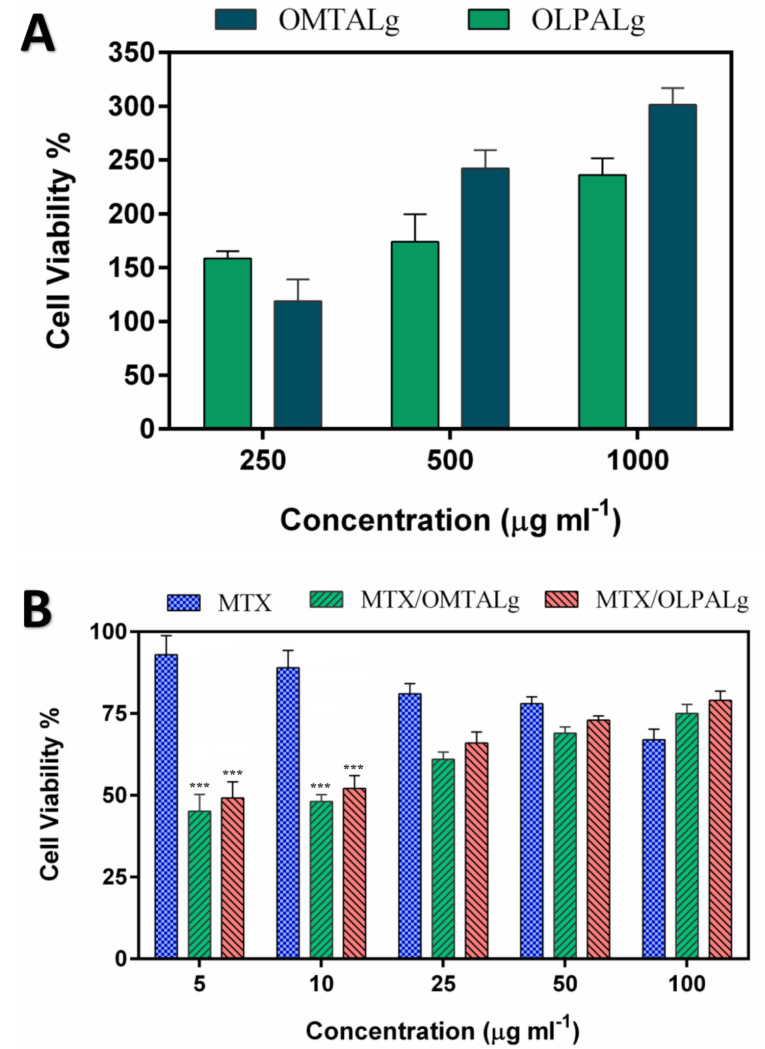
Cell growth inhibition rates by (A) different concentrations OMTALg and OLPALg nanocomposites, (B) free MTX, MTX-loaded OMTALg (MTX/OMTALg), and MTX-loaded OLPALg (MTX/OLPALg).

MCF-7 cells viability studied by MTT assay method showed that the administration of both MTX alone or in nano-formulation form increased mortality rate compared to non-treated cells (Figure 7). Interestingly, MCF-7 cells treated with MTX loaded OLPALg and OMTALg nanocomposites (concentration of 5 and 10 µg mL-^1^) significantly increased cell mortality compared to cells treated with free MTX (*P*_ value_<0.001). Several studies revealed a protective effect of clay surfaces on cancer cells, stem cells and primary cells.^30,55-57^ For example, a decrease in toxicity of anticancer drug to human neuroblastoma cells was reported when using 6-mercaptopurine loaded bentonite clay.^58^

A key item in drug carrier polymers is their biocompatibility which can be assessed by outcomes of MTT assay. Our results indicate that OLPALg and OMTALg nanocomposites were nontoxic to MCF-7 cells as shown in Figure 7A. Excellent biocompatibility of montmorillonite/chitosan nanocomposites was reported by Hsu and colleagues.^59^ In another study, long term bio-compatibility of alginate encapsulated particles in primates was mentioned.^60^ Biocompatibility of laponite clay formulations were shown by Tzitzios et al.^61^ These studies are in accordance with our findings which highlight nontoxicity and even proliferative effects of OLPALg and OMTALg nanocomposites on MCF-7 cell line.

### Cell cycle assay

**Figure 8:**
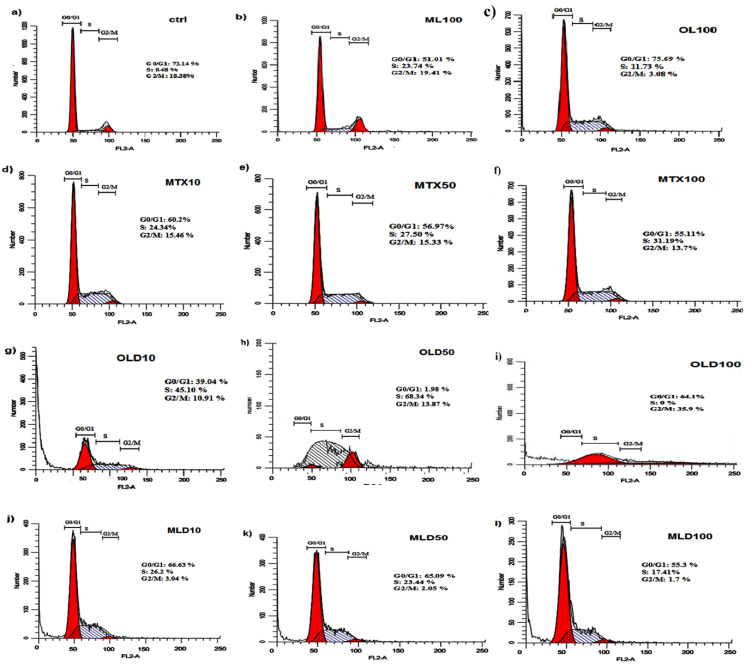
The cell cycle analysis graphs in a) control, b) 100μg.mL-^1^ ML, c) 100 μg.mL-^1^ OL, (d) 10 μg.mL-^1^ MTX, e) 50 μg.mL-^1^ MTX, f) 100 μg.mL-^1^ MTX, g) 10 μg.mL-^1^ OLD, h) 50 μg.mL-^1^ OLD, i) 100 μg.mL-^1^ OLD, j) 10 μg.mL-^1^ MLD, k) 50 μg.mL-^1^ MLD and l) 100 μg.mL-^1^ MLD obtained by flowCytometry (OL: organo-modified Laponit-RD composite, ML: organo-modified montmorillonite, MTX:Methotrexate, OLD:MTX-loaded OL, MLD:MTX-loaded ML).

**Figure 9:**
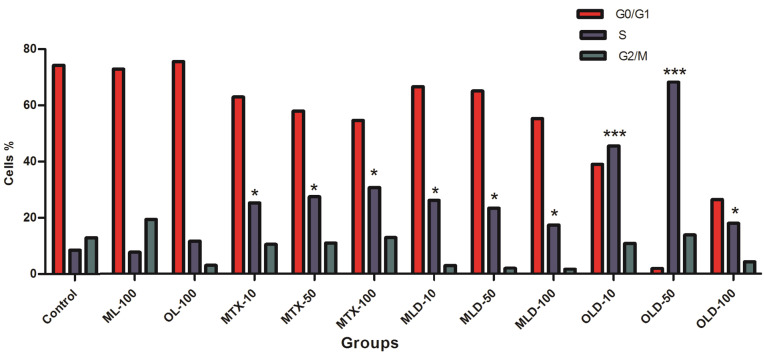
The frequencies of the cells in cell cycle phases. Higher S-phase arrest for OLD compared to free MTX. * *P* ˂ 0.05, ***P ˂ 0.01.

The effect of free MTX, MTX-loaded OLPALg and OMTALg nanocomposites were investigated on MCF-7 cell cycle arrest. Our results showed that MTX treated groups has arrested MCF-7 cells in S-phase (Figures 8d, e, f) in comparison with non MTX-treated groups (Figures 8a, b, c). The results indicated that the MTX-free nanocomposites have no significant disturbing effect on cell cycle (Figures 8b, c). No disturbing effect of alginate, montmorillonite and laponite on cell cycle has been proved previously. For example, encapsulation of cells in alginate polymers had no reported effect on mesenchymal stem cells from various origins.^62^ Our previous work on clay-based nanocomposites showed no disturbing effect on MCF-7 cell cycles.^40^ Although, the results emphasized on non-toxicity of developed nanocomposites, the MTX-loaded nanocomposites (Figures 8g-l) caused significant accumulation of cells in S-phase in both OLD (MTX loaded OLPALg) (Figures 8 g, h, i) (*P* < 0.001) and MLD (MTX loaded OMTALg) (Figures 8j, k, l) (*P* < 0.05). These findings clearly showed higher anti-cancer effects of MTX-loaded nanocompositions. Dose dependency in OLD was obvious and MTX-loaded polymers exhibited higher toxicity at higher doses (Figure 9). In general, our results confirmed that MTX-loaded OLPALg caused higher interruption on cell cycle compared to MTX-loaded OMTALg.

## Conclusion

An innovative polymeric nano-composite with average particle size below 70 nm was engineered using alginate, clay, and imidazolium-based ionic liquid. Intercalation of alginate and imidazolium based ionic liquid into the organo modified Laponite RD and montmorillonite was confirmed by widening of d-spacing in XRD spectra. CIP encapsulated polymer/clay nanocomposites showed better inhibitory effects on *E. coli* compared to free CIP. These synthesized nanocomposites with antimicrobial effects have high potential in scientific research as drug delivery systems in cancer therapy. The dual MTX-CIP formulated smart OLPALg and OMTALg nanocomposites provided the opportunity for co-delivery of both MTX and CIP drugs with increased release in tumor tissue. To conclude, results of this study confirmed that dual drug conjugated polymer/clay nanocomposites are promising tools for cancer combination chemotherapy.

## Conflict of Interest

The authors declare that there are no conflicts of interest.

## Ethical Issues

Not applicable.
